# Strain-tunable triple point Fermions in diamagnetic rare-earth half-Heusler alloys

**DOI:** 10.1038/s41598-021-90850-y

**Published:** 2021-06-08

**Authors:** Anupam Bhattacharya, Vishal Bhardwaj, Brajesh K Mani, Jayanta K Dutt, Ratnamala Chatterjee

**Affiliations:** 1 Department of Mechanical Engineering, Indian Institute of Technology Delhi, New Delhi, India; 2 Department of Physics, Indian Institute of Technology Delhi, New Delhi, India

**Keywords:** Topological matter, Density functional theory, Electronic structure, Topological matter

## Abstract

Topologically non-trivial electronic structure is a feature of many rare-earth half-Heusler alloys, which host atoms with high spin-orbit coupling bringing in the non-triviality. In this article, using the first-principles simulations, rare-earth half-Heusler YPdBi, ScPdBi, LaPdBi, LuPdBi, YPtBi and LuPtBi alloys are studied under strain to reveal multiple band inversions associated with topological phase transitions. From our simulations we find that, as a result of first band-inversion, the Brillouin zone of the diamagnetic half-Heusler alloys hosts eight triple points whereas, the second band inversion causes the emergence of sixteen more triple points. These band-inversions are observed to be independent of the spin-orbit coupling and are the reason behind increasing occupation of bismuth 7*s* orbitals as volume of the unit cell increases. The surface electronic transport in different triple point semi-metallic phases is found to evolve under strain, as the number of Fermi arcs change due to multiple band inversions. Once the second band inversion occurs, further application of tensile strain does not increase the number of triple points and Fermi arcs. However, increasing tensile strain (or decreasing compressive strain) pushes the triple point crossing to higher momenta, making them more effective as source of highly mobile electrons. These observations make a pathway to tune the bulk as well as surface transport through these semi-metals by application of tensile or compressive strain depending on the unstrained relative band-inversion strength of the material.

## Introduction

Half-Heusler alloys have come to the limelight in the area of novel materials research as they have shown evidence of multiple new functionalities such as antiferromagnetic spintronics^1–3^, thermo-electric effects^4–7^, topological non-triviality of electronic bands^8–10^, etc. Multiple members of rare-earth half-Heusler family, represented by a common chemical formulae RYZ (where, R = rare-earth elements, Y = Pd/Pt and Z = Bi/Sb), have shown topological non-triviality in their band-structures^11,12^. The origin of this topological character is attributed to their HgTe like lattice structure^13^, which allows it to have single point proximity of conduction and valence bands at $\Gamma$ point; whereas, these bands stay apart elsewhere in the Brillouin Zone (BZ). The valence bands are predominantly of *p* and *d* characters and the conduction bands are of *s* character. Under relativistic effects, the valence bands spilt in energy and rise above the *s* type conduction bands creating a band inversion at $\Gamma$ point. This band inversion is the key for obtaining topological non-triviality in these materials^14^.

**Figure 1:**
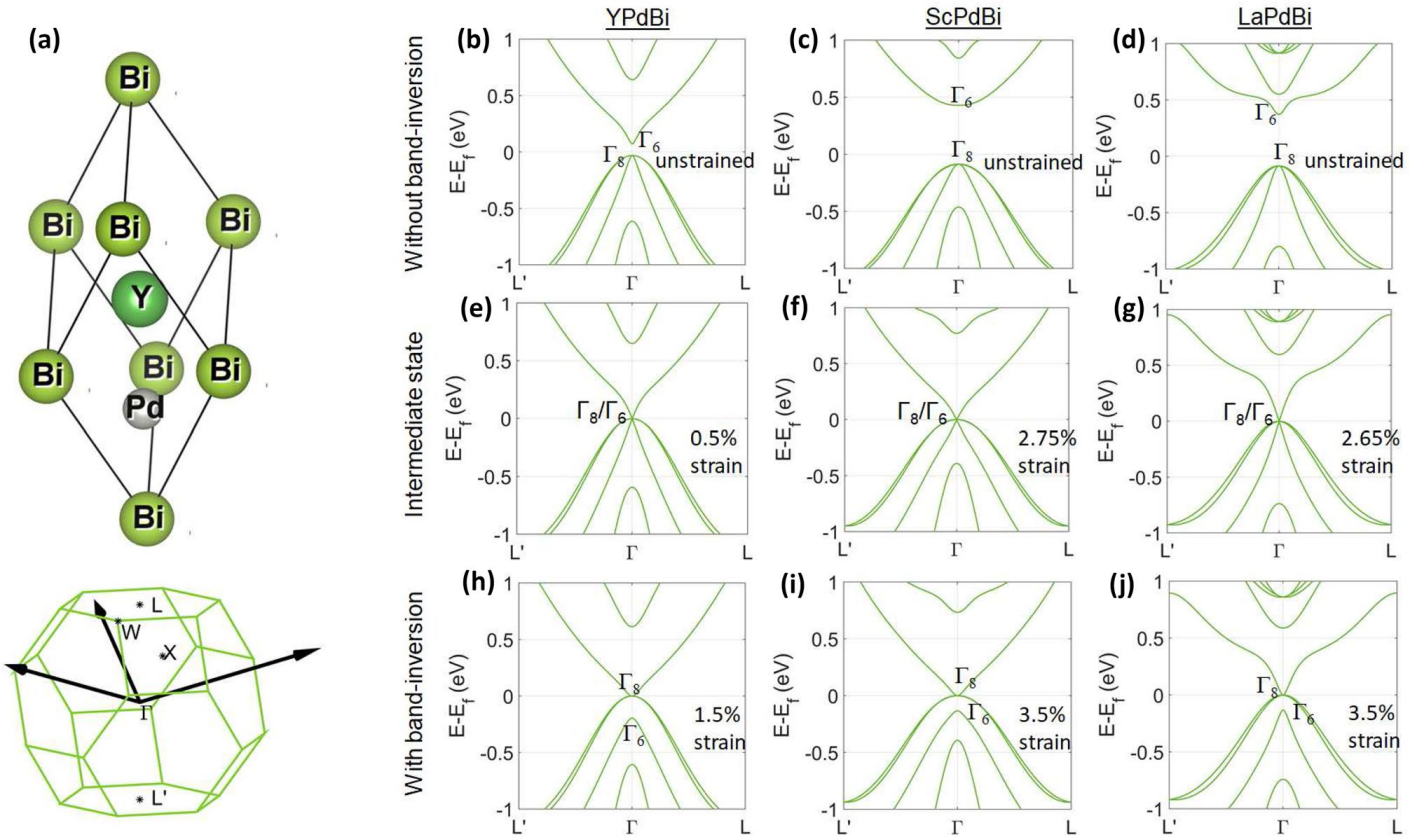
(**a**) Primitive unit cell of YPdBi and corresponding BZ with high-symmetry points and reciprocal vectors. All the materials considered in the present work has the same unit cell structure. (**b**–**j**) Band structure in YPdBi, ScPdBi and LaPdBi as a function of uniform tensile strain. Similar data for LuPdBi, YPtBi and LuPtBi under compressive strain are presented in the supplemetary information.

The diamagnetic rare-earth half-Heusler alloys such as YPtBi, YPtSb, LuPtBi, LaPtBi, etc. show band inversion, making them topologically non-trivial. Moreover, in a recent theoretical study^15^, a prediction of topological semimetallic states hosting triple point fermions were reported in these non-trivial materials. In contrast, in some of the rare-earth half-Heusler alloys like YPdBi, ScPdBi and LaPdBi, the relativistic splitting itself is not sufficient to create a band-inversion^16–18^. These semiconductors are, however, reported to show a band-inversion at $\Gamma$ point under uniform tensile strain^19^. These findings invite further studies on strained-induced topological phase transition in half-Heusler alloys, which can provide answers to the following questions: (1) can rare-earth diamagnets such as YPdBi, ScPdBi and LaPdBi host topolologically protected Fermions? (2) what is the mechanism associated with this strain-induced phase transition in half-Heusler alloys? (3) can there be multiple band inversions? (4) how does the topologically protected triple-point Fermions evolve under strain?, and (5) how does this strain-induced transition affect the bulk and surface conductivities in these materials?

In this study, with the help of first-principles simulations, we aimed to address these questions. The electronic structures of strained YPdBi, ScPdBi, LaPdBi, LuPdBi, YPtBi and LuPtBi are analyzed to demonstrate multiple band inversions and trace the mechanism associated with it. Emerging triple-point fermions, their evolution and resulting topological phases as function of uniform strain are examined in detail. Moreover, the change in the nature and the number of surface Fermi arcs originating from these triple points under different strains is also reported. From our simulations we observed that the strain tuned diamagnetic rare-earth half-Heusler alloys can obtain different triple point semi-metallic phases, which provides a mechanism to control the bulk and surface electronic transport through the materials.

## Results

**Figure 2:**
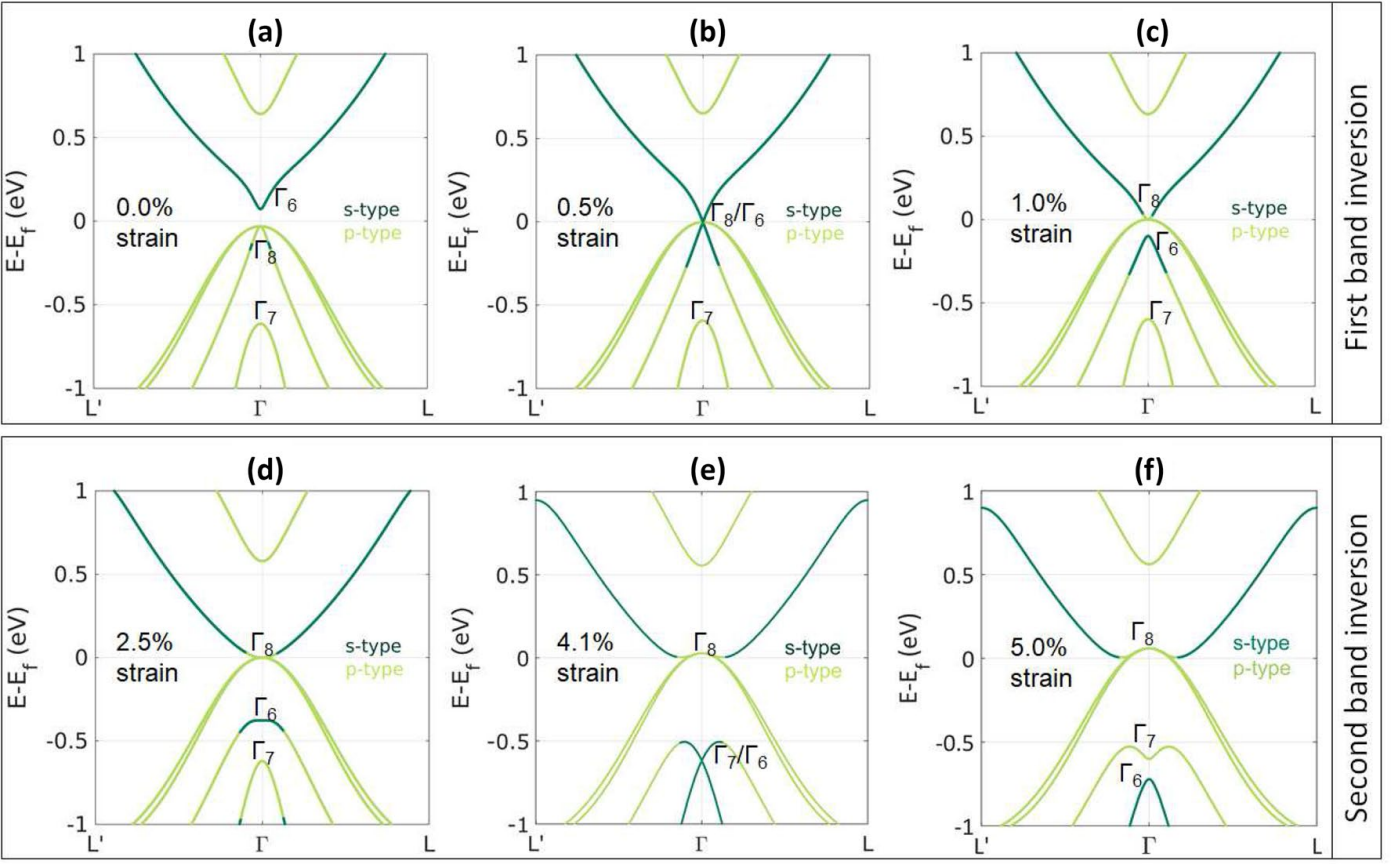
Electronic band-structure of YPdBi under uniform tensile strain showing two band inversions. Dark green and light green colors represent *s* type and *p* type bands respectively.

### Tensile strain induced band-inversion

The half Heusler alloys have FCC lattice structure with a space group $F{\overline{4}}3m$. In diamagnetic R(Pd/Pt)Bi, R, Pd/Pt and Bi occupy the wyckoff positions (0.5,0.5,0.5), (0.25,0.25,0.25) and (0.0,0.0,0.0), respectively. Figure 1(a) shows the rhombohedral primitive unit cell for which $a=b=c=a_{\mathrm{cubic}}/\sqrt{2}$ and the corresponding BZ with high symmetry points.

In Figure 1, we have shown the electronic band structures of YPdBi, ScPdBi and LaPdBi with and without tensile strains. These electronic structures are computed using the equilibrium lattice constants which are obtained by minimizing the total free energy as a function of lattice constants. Calculated lattice parameters for YPdBi, ScPdBi and LaPdBi are 6.570Å, 6.365Å and 6.788Å, respectively, which are in good agreement with the reported experimental values 6.64Å, 6.43Å and 6.82Å, respectively^20^. The small differences from experimental values are attributed to the use of LDA pseudopotentials in our calculation.

Figures 1(b-d) show the electronic band structures of YPdBi, ScPdBi and LaPdBi in unstrained state. Clearly, none of the electronic structures show the band inversion, with two-fold degenerate $\Gamma _6$ higher in energy than the four-fold $\Gamma _8$. The calculated band inversion strengths ($E_{\Gamma _8}-E_{\Gamma _6}$) for these materials are -0.103, -0.516 and -0.456 eV, respectively. This indicates that, among these three materials, YPdBi has the least negative band inversion strength, and therefore, is expected to require less tensile strain to undergo band inversions than ScPdBi and LaPdBi. A similar trend of the band inversion strength is also observed in the experimental studies^17,18^. Band-structure calculations of YPdBi, ScPdBi and LaPdBi with HSE06 functional were also performed to validate the LDA predicted band gaps, and are given in the supplementary information. Figures 1(e–g) show the onset of band inversion when $\Gamma _8$ and $\Gamma _6$ coincide. These intermediate states are observed for 0.5%, 2.75% and 2.65% tensile strains in YPdBi, ScPdBi and LaPdBi, respectively. The complete band inversions are achieved with 1.5%, 3.5% and 3.5% of uniform tensile strain in YPdBi, ScPdbi and LaPdBi, respectively. This is shown in the Figures 1(h-j), where the four-fold degenerate $\Gamma _8$ lies above the two-fold $\Gamma _6$. Other three materials studied in this work viz. LuPdBi, YPtBi and LuPtBi have band inversion in the unstrained state and the corresponding data is given in the supplementary information.

### Band inversion mechanism

Since YPdBi shows the least negative band-inversion strength, for further studies on the mechanism associated with strain-induced band inversion, we focus on YPdBi. Similar descriptions for other materials considered in this work are given in the supplementary information. To visualize the band inversion, *s* and *p*-characters were assigned to bands^21^ and plotted as function of different tensile strains in Figure 2. Under no strain, *s*-type $\Gamma _6$ lies above $p_{3/2}$-type $\Gamma _8$ and $p_{1/2}$-type $\Gamma _7$. At 0.5% strain, the kink in the conduction bands (Figure 2(a)) extends to the Fermi energy and connects with the valence bands (Figure 2(b)). Out of the two pairs of valence bands, one with larger radius of curvature conserves its *p*-character. The orbital *s*-character from the conduction band bleeds into the high curvature valence band pair through the kink. This stage demonstrates how orbital character changes during band-inversion and is referred as the intermediate state. At 1% tensile strain (Figure 2(c)), the *s*-character in the valence bands gets disconnected from the conduction bands. At this stage, the band-inversion is complete, as the $p_{3/2}$-type $\Gamma _8$ lies above the *s*-type $\Gamma _6$.

**Figure 3:**
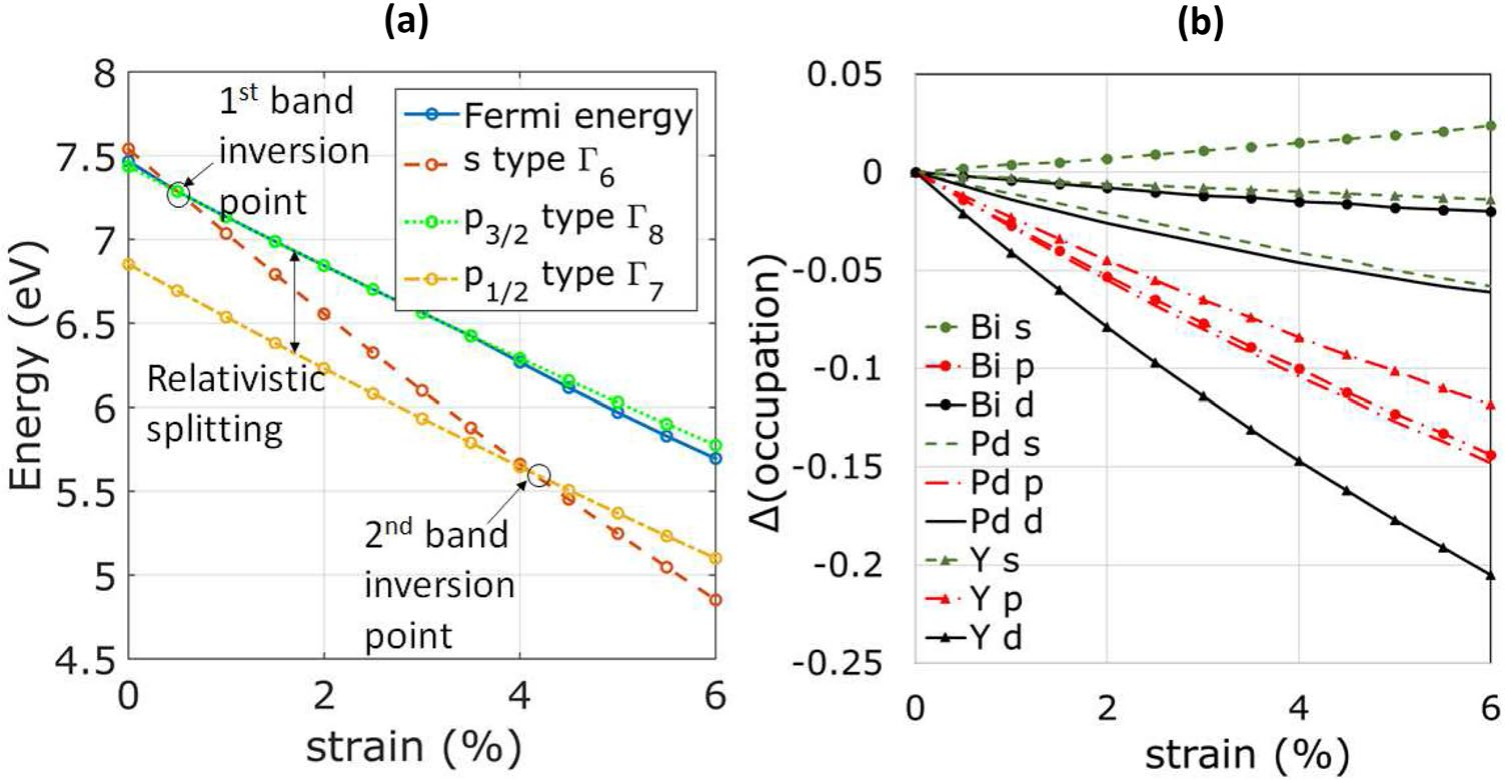
(**a**) Change in Fermi energy, $\Gamma _6$, $\Gamma _8$ and $\Gamma _7$ with tensile strain. (**b**) Change in electronic occupation from unstrained state.

As the tensile strain is further increased, another band-inversion takes place. At 2.5% strain (Figure 2(d)), a small downward kink develops at $\Gamma _6$. Higher tensile strain further pulls $\Gamma _6$ downwards until it meets $\Gamma _7$ approximately at 4.11% tensile strain. Here, interestingly, again *s*-character bleeds from upper valence bands into the lower valence bands through a kink. This second band-inversion is complete when *s*-type $\Gamma _6$ goes below $p_{1/2}$-type $\Gamma _7$ (Figure 2(f)). The maximum value of tensile strain (5%) explored in the present study is kept limited by the experimental values applicable to thin films. Recent experimental studies on thin films of rare-earth half Heusler alloys^22–24^ have shown that up to 3.12% tensile strain could be achieved in (110) oriented thin films without distortion of the cubic symmetry. Therefore, it is expected that in future experiments, possibility to accommodate higher values of tensile strain will be explored.

To get further insight to the band inversion mechanism, the energies of $\Gamma _6$, $\Gamma _8$, $\Gamma _7$ and Fermi level are plotted as a function of strain in Figure 3(a). All the energies decrease linearly with the increasing strain. However, the gap between $\Gamma _8$ and $\Gamma _7$ (the vertical distance between the green and yellow lines as shown by the arrow), which is a measure of the relativistic splitting of *p*-orbitals, remains constant in the simulated range. This implies that uniform strain does not affect the spin-orbit coupling and hence spin-orbit coupling is not the reason for strain-induced band inversions in these materials. Interestingly, the *s*-type $\Gamma _6$ decreases in energy much faster than *p* bands, enabling the two band inversions. This trend of rapidly changing $\Gamma _6$ and change in occupation of *s*-orbital in Bi is observed for all the materials studied. (corresponding data for LuPdBi, YPtBi and LuPtBi are presented in supplementary information).

Figure 3(b) shows the change in the orbital occupations as the crystal is strained. Clearly, only bismuth *s*-orbital occupation increases with increasing strain. Since, the total number of valence electrons is constant in the system, this implies that, with increasing volume, more electrons occupy the regions outside Muffin-tin radii of the atoms, and as a result, the bismuth 7*s* occupation increases at the cost of mostly *d*-orbitals from Yttrium and *p*-orbitals from all the atoms.

### Triple point semi-metallicity

**Figure 4:**
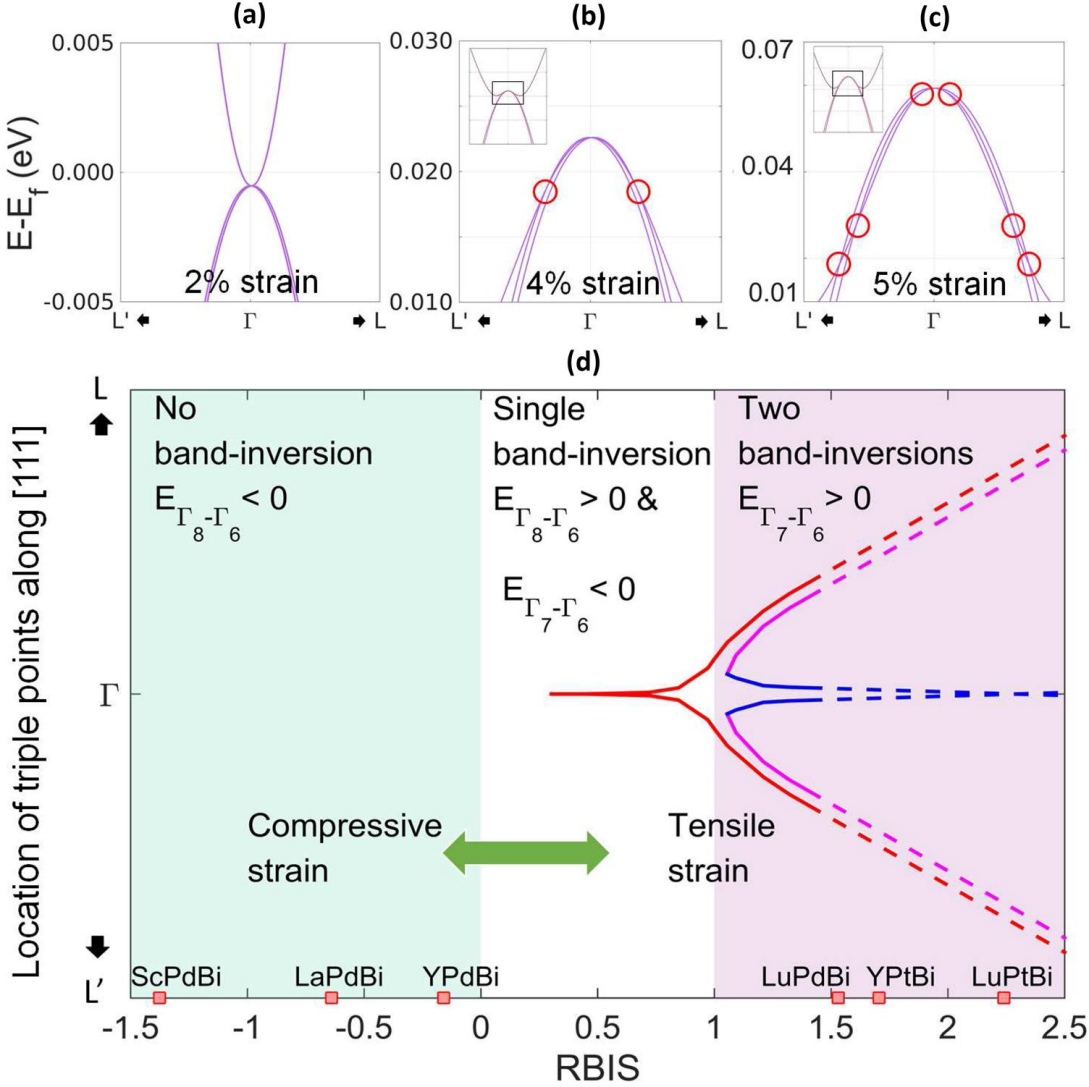
Triple points (marked with red circles) in YPdBi at (**a**) 2%, (**b**) 4% and (**c**) 5% tensile strain. (**d**) Trajectories of triple points in diamagnetic half-Huesler alloys as function of RBIS. The red, magenta and blue lines represent the trajectories of the outer-most, middle and inner-most triple points respectively.

Next, we study the topological semi-metallicity in detail in diamagnetic rare-earth half-Heusler alloys in the light of multiple band inversions observed so far. Topological semimetals have linear electronic dispersion in their BZ and are broadly classified based on the degeneracy at these crossing points. The triply degenerate topologically protected nodes or triple points are predicted to exist in several materials^25–29^ in the last few years. These triple points are protected by coexistence of both three-fold rotational symmetry and vertical mirror symmetries (together referred to as $C_{3v}$ symmetry). In rare-earth half Heusler alloys, triple points were predicted in the recent study by Yang *et. al.*^15^. These materials belong to the $F{\overline{4}}3m$ space group and has $T_{d}$ point group and therefore fulfills the necessary symmetry conditions for hosting triple point fermions.

**Table 1:** Number and momenta of triple points in YPdBi for different values of tensile strain.

Tensile strain (%)	Total number of triple points in BZ	Location of triple points (reciprocal coordinates)	Energy w.r.t. Fermi energy (eV)	Band inversion
1.5–2.5%	8, Indistinguishable as very close to $\Gamma _8$	$\approx$(0.0, 0.0, 0.0)	0.000	single band inversion
3%	2 along each <111> of FCC BZ; total 8	$(\pm 0.001,\pm 0.001,\pm 0.001)$	0.000	
3.5%	8	$(\pm 0.004,\pm 0.004,\pm 0.004)$	0.002	
4.0%	8	$(\pm 0.017,\pm 0.017,\pm 0.017)$	0.018	
5.0% and beyond	6 along each <111> of FCC BZ; total 24	$(\pm 0.006,\pm 0.006,\pm 0.006)$ $(\pm 0.045,\pm 0.045,\pm 0.045)$ $(\pm 0.054,\pm 0.054,\pm 0.054)$	0.058 0.027 0.018	two band inversions

In order to look for linear triple point like dispersion, the electronic structure of inverted YPdBi is analyzed further around the $\Gamma$ point where the conduction and valence bands touch. The corresponding data for ScPdBi, LaPdBi, LuPdBi, YPtBi and LuPtBi is given in the supplementary information. Figure 4 shows the electronic structure of non-trivial YPdBi in the vicinity of Fermi energy and $\Gamma$ point for different strains. As a result of the point group $T_d$, the *s*-type conduction bands remain doubly degenerate while the *p*-type valence bands become non-degenerate away from $\Gamma _8$ point. Therefore, any crossing apart from $\Gamma _8$ between the conduction and valence bands inevitably becomes triply degenerate. For tensile strain in the range $0.5\% < \epsilon \le 2.5\%$, the conduction and valence bands have low radii of curvature. The electronic band structure of YPdBi in such state (2% tensile strain) is shown in the Figure 4(a). In this range of strain, the conduction and the valence bands cross very close to $\Gamma _8$ point, making the triply degenerate nodes indistinguishable from $\Gamma _8$. With increasing tensile strain ($\epsilon > 2.5\%$), curvature of the conduction bands at $\Gamma$ decreases from highly positive values (Figure 4(a)) to negative values creating a double well structure of the bands (Figures 4(b) and (c)), thereby taking the crossing point away from $\Gamma _8$. As figure 4(b) shows, at 4% strain, the conduction bands create two triply degenerate crossings along [111]. Therefore, within the range $0.5\%<\epsilon < 4.1\%$ (where YPdBi hosts a single band inversion) two triple point crossings emerge along each [111], which leads to eight triple points in the BZ (Table 1).

As observed in Figure 4(c) for 5% tensile strain, increasing the strain further ($\epsilon > 4.1\%$) makes the conduction bands cross the other valence band, creating two more pairs of triple points along each [111] axis. Clearly, a topological phase transition takes place as a result of the second band inversion (as observed in the previous section) changing the number of topologically protected nodes in the BZ. Further discussions related to this observed topological transition based on $Z_2$-invariants scheme is presented in the next section. Increasing the strain beyond 5% further reduces the curvature of the conduction bands, however, the number of crossings remain unchanged. The momenta of the observed triple point Fermions at different tensile strains for YPdBi are given in the Table 1. And, for a better visibility, the trajectories of triple point Fermions along the $\Gamma - L$ k-line is shown in the Fig. 4(d). As discernible from the figure, we observe a trend of nonlinear change in the momenta with strain. Similar trends are also observed for other half-Heusler alloys studied in this work.

The phase diagram shown in Figure 4(d) summarizes the emergence and evolution of triple points which can be tuned by applying suitable tensile or compressive stains depending on the unstrained phase of the material. Since the relativistic splitting, $E_{\Gamma _8-\Gamma _7}$, remains constant for each material under strain (Figure 3(a)), in order to normalize the band-inversion strengths of materials, we define the *relative band-inversion strength* (RBIS) of the materials,

1 $$\begin{aligned} \mathrm{RBIS} = \frac{E_{\Gamma _8-\Gamma _6}}{E_{\Gamma _8-\Gamma _7}}. \end{aligned}$$

As a result of this normalization, the first and second band-inversions occur at $\mathrm{RBIS}=0$ and 1, respectively in each material. Under no strain, the dimagnets YPdBi, LaPdBi and ScPdBi lie in the zone where no triple points are observed. These materials can be tuned with the help of tensile stain to exhibit the first band-inversion where the emergence of triple points takes place. Further applications of tensile strains leads to the second band-inversion where more triple points emerge and the Fermions drift away from the $\Gamma$ point. These drifted Fermions can be easily detected using angle-resolved photo-emission spectroscopy. This tunability can also be achieved through the compressive strains in the materials which lie in the second band-inversion zone in unstrained state. For example, as shown in Figure 4(c), LuPdBi, YPtBi and LuPtBi exhibit two band inversions and host 24 triple points in unstrained state. A similar observation was reported in a study by Yang *et al.*^15^. As shown in the phase diagram, calculated values of RBISs for these materials are 1.53, 1.71 and 2.24, respectively. Supporting data on electronic band structure for LuPdBi, YPtBi and LuPtBi are given in the supplementary information.

### Surface electronic structure

Next we focus on the change in the surface transport properties as a result of topological change in diamagnetic half-Heusler alloys. The electronic transport due to the triple point crossings are mediated largely by Fermi arc like surface states^30^, which are the signatures of Weyl and triple-point fermions. A triple-point Fermion, which is equivalent to two degenerate Weyl fermions of opposite chirality and located at the same k-point because of spatial symmetry, acts as the origin of two Fermi arcs^15^. Using the Maximally-Localized Wannier Function (MLWF) based tight binding model, a semi-infinite slab of YPdBi terminated along (111) surface is simulated to study the surface electronic structure.

**Figure 5:**
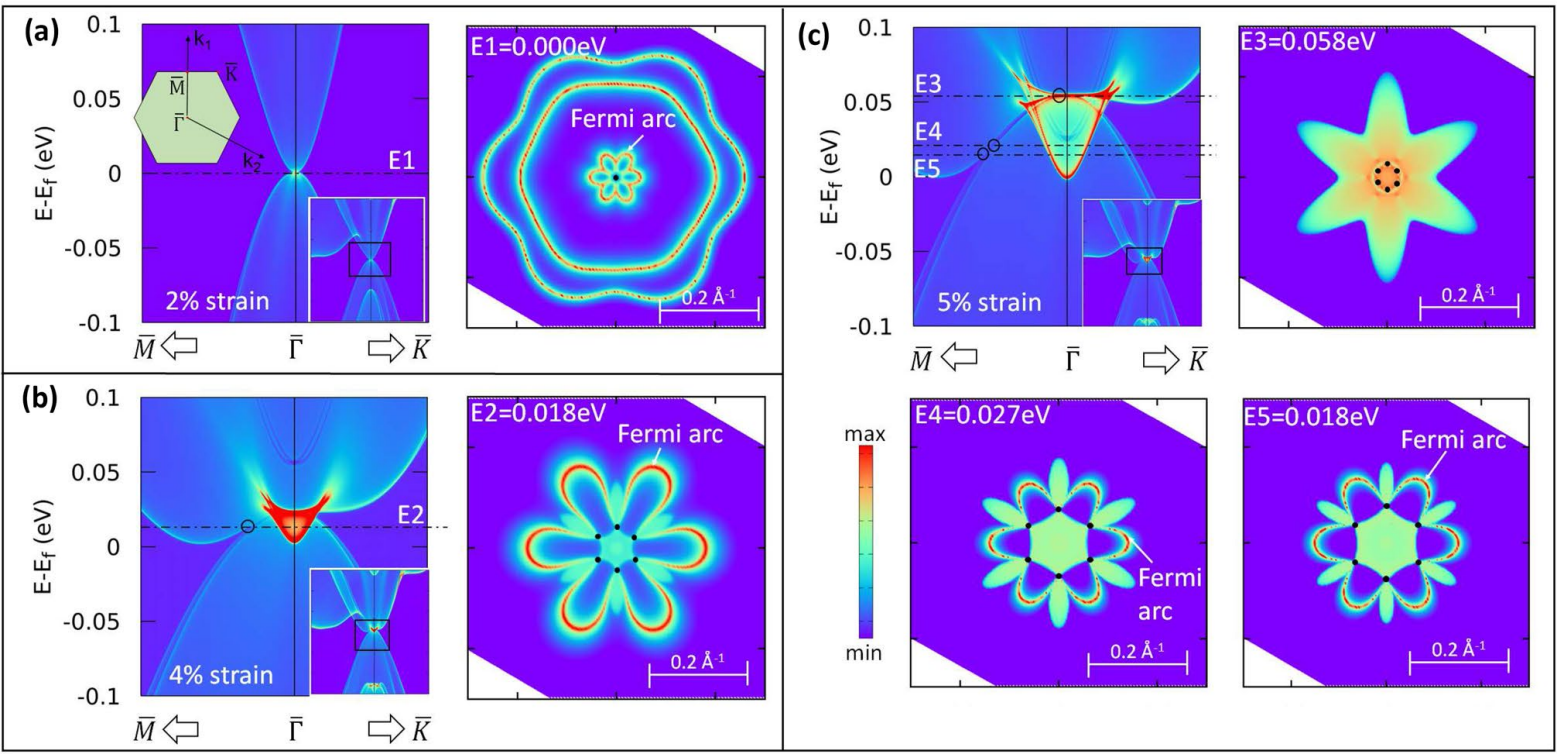
Electronic structure of semi-infinite slabs of YPdBi under (**a**) 2% (**b**) 4% and (**c**) 5% tensile strains. Inset in (**a**) shows 2D BZ of the semi-infinite slab with high symmetry points.

For this, 2%, 4% and 5% strained YPdBi slabs are simulated. The K-resolved bulk DOS for 2% strained YPdBi, Figure 5(a-left), is reminiscent of the bulk electronic structure shown in Figure 4(a). Since the crossings are located very close to the ${\overline{\Gamma }}$, the iso-energy electron density (to the right) on the (111) surface shows flower like surface states all emerging from ${\overline{\Gamma }}$. The electronic dispersion under 4% strain (Figure 5(b-left)), along ${\overline{M}}-{\overline{\Gamma }}$ line is again similar to the bulk dispersion shown in Figure 4(b). The zoomed view of Figure 5(b) shows the locations of the triple points marked by a hollow circle. The iso-energy electronic density calculated at 0.018eV (E2) is shown in the Figure 5(b-right). Here, the black dots represent the locations of the triple points and the two red colored Fermi arcs emerging from each triple point forms the flower petal like shape.

The electronic structure of the 5% strained semi-infinite slab is shown in the Figure 5(c). The K-resolved density of states shown in Figure 5(c-left) reveals three crossings along ${\overline{M}}-{\overline{\Gamma }}$, similar to the bulk electronic structure discussed in previous subsection. These crossings are observed at 0.058 eV (E3), 0.027 eV (E4) and 0.018 eV (E5). The iso-energy electronic density calculated at these energies are shown in Figure 5(c). The distinct Fermi arcs are visible for triple points at 0.018eV and 0.027eV. However, Fermi arcs could not be identified for triple points at 0.058eV due to dense bulk background. When compared with figure 5(b), it becomes evident that, the second band inversion not only gives rise to the number of triple-points but increases the number of associated Fermi arcs, thereby increasing the surface transport in the slab.

## Discussion

We have carried out density functional theoy based first-principles study on the strain-induced topological non-triviality in rare-earth half-Heusler YPdBi, ScPdBi, LaPdBi, LuPdBi, YPtBi and LuPtBi alloys. The discussions on our study can be divided into the following parts.

First, we find that the YPdBi, ScPdBi and LaPdBi (LuPdBi, YPtBi and LuPtBi) undergo multiple band-inversions under varying tensile (compressive) strain. Among YPdBi, ScPdBi and LaPdBi, the YPdBi is the most promising candidate for introducing the topological semi-metallicity with tensile strains. From the detailed analysis of the electronic structure of these materials we find that, opposite to the trends for other electrons, the occupation of the unfilled 7*s* orbital of bismuth increases, leading to a more rapid decrease in the energy of $\Gamma _6$ than $\Gamma _7$ and $\Gamma _8$, with the increasing volume of the unit cell.

Second, topologically protected triple-point fermions are observed in the BZ of strained lattice of rare-earth half-Heulser alloys. Eight triple points emerge in the BZ as a result of the first band inversion. The second band inversion brings in 16 more triple point crossings through another topological phase transition. To support our view on the relation between emergence of triple points and band inversions, we followed a $Z_2$ invariant based scheme. The $Z_2$ invariants were calculated on time-reversal invariant $k_i=\{0.5\}$ planes, where $i=1, 2, 3$. Since these planes are the boundary planes of the truncated-octahedra Brillouin zone of the $F{\overline{4}}3m$ space group and are perpendicular to the reciprocal lattice vectors, $Z_2$ invariants at these planes do not necessarily imply the topological triviality of the material. However, $Z_2$ invariants can change when a nontrivial band crossing takes place on the corresponding 2D plane. Thus, the change in $Z_2$ invariants indicates that there is a change in the triple points and corresponding surface states^31^. We have observed that in the case of no band inversion, the $Z_2$ invariants are each zero. After the first band inversion, the $Z_2$ invariants change to 1. This leads to the emergence of 8 triple points. And, as a result of second band inversion, the $Z_2$ invariants change back to zero and, interestingly, this change happens immediately after the second band inversion. It is to be however emphasized that the material is still in the nontrivial state. To explain the connection between the second band inversion and the triple points, we refer to the phase diagram in the Figure 4(d). The figure shows that 16 more triple points become visible at a certain RBIS value above 1 (second band-inversion happens at $\mathrm{RBIS}=1$). In this zone, where the second band inversion has taken place but the 16 more triple points are not visible in the band-structure, surprisingly the $Z_2$ invariants seemed changed from 8 triple point state. For example, we studied 4.2% tensile strained YPdBi (second band inversion took place at 4.11% tensile strain and 24 triple points were visible from 4.3% tensile strain), which lies in this zone and $Z_2$ invariant for this strained state on all $k_i=0.5$ planes are zero, which has changed from 1 for the 8-triple point case (4.1% strain) . We also see that the $Z_2$ invariants remain same in the whole second band inverted region. This implies that although more number of triple points are not recognizable in the band-structure, they exist and have emerged along with the second band-inversion.

And third, the bulk conductivity is expected to increase in these materials due to the increase in the number of the triple points. Moreover, the triple points drift away from the $\Gamma$ to higher momenta as a result of increasing tensile (decreasing compressive) strain. In addition, since each of these crossings gives rise to two Fermi arcs, the surface electronic transport is also expected to increase. This provides a way to control and tune the surface transport properties through tensile (compressive) strain in YPdBi, ScPdBi and LaPdBi (LuPdBi, YPtBi and LuPtBi) half-Heusler alloys.

## Methods

Fully relativistic first-principles calculations are carried out using the density functonal theory (DFT) as implemented in Vienna Ab-initio Simulation Package (VASP)^32–35^. The projector augmented-wave (PAW)^36,37^ pseudopotentials with a plane wave basis with energy cutoff of 400 eV are used in all the calculations. The exchange-correlation among the electrons is incorporated using local density approximation (LDA) as formulated by Ceperley and Adler^38^. The convergence criterion used for all self-consistent field calculations is $10^{-8}$ eV per formula unit. All the strained structures are relaxed until atomic forces reduce below $5\times 10^{-5}$ eV/Å. The Monkhorst-Pack^39^ k-mesh of $13\times 13\times 13$ is used for 3D periodic bulk calculations. A Maximally-Localized Wannier Function (MLWF) based tight binding model as implemented in Wannier90^40,41^ is used to calculate the electronic structure of semi-infinite slabs.

## Supplementary Information

Supplementary Information.

## Data Availability

The data generated during and/or analysed during the current study are available from the corresponding author on reasonable request.
