# Author Correction: Strain-tunable triple point Fermions in diamagnetic rare-earth half-Heusler alloys

**DOI:** 10.1038/s41598-021-95766-1

**Published:** 2021-08-04

**Authors:** Anupam Bhattacharya, Vishal Bhardwaj, Brajesh K. Mani, Jayanta K. Dutt, Ratnamala Chatterjee

**Affiliations:** 1 Department of Mechanical Engineering, Indian Institute of Technology Delhi, New Delhi, India; 2 Department of Physics, Indian Institute of Technology Delhi, New Delhi, India

Correction to: *Scientific Reports* https://doi.org/10.1038/s41598-021-90850-y, published online: 08 Jun 2021

The original version of this Article contained an error in the Acknowledgments section.

"Authors R. Chatterjee, A. Bhattacharya and V. Bhardwaj acknowledge the financial support received from the SPARC project #754 (RP03738G), funded by MHRD, India".

now reads:

"RC, AB and VB acknowledge the financial support received from the DST-Nanomission project DST/NM/TUE/QM-11/2019 G (RP03993G), India."

The original Article has been corrected.

