# Supplementary material for: Strain-tunable triple point Fermions in diamagnetic rare-earth half-Heusler alloys
Source: Sci Rep. 2021 Jun 8;11:12029. doi: 10.1038/s41598-021-90850-y (PMC8187712; doi:10.1038/s41598-021-90850-y)
Supplement: Supplementary file 1 — Supplementary Information. [file 41598_2021_90850_MOESM1_ESM.pdf]

Supplementary Information: Strain-tunable triple point Fermions in diamagnetic rare-earth half-Heusler alloys

Anupam Bhattacharya, Vishal Bhardwaj, Brajesh K Mani, Jayanta K Dutt and Ratnamala Chatterjee

Band inversion and triple points in ScPdBi and LaPdBi through tensile strain

Both ScPdBi and LaPdBi are semiconductors without band inversion in unstrained state (Figure 1(a)). As shown in the $s-p$ projected bands in Figure 1(b-c), at 5% tensile strain, ScPdBi hosts two triple points along each [111] direction, and hence lies in the *single* band-inversion zone of the phase diagram (Figure 4(d) in the manuscript). To realize the triple points away from the Γ_8, we need to apply more tensile strain than what is required for the first band-inversion. And, as shown in Figure 1(d-e), ScPdBi is observed exhibits six triple points along each [111] with *two* band-inversions at 8% tensile strain.

For LaPdBi, the value of tensile strain required for realizing triple points is observed to be more than ScPdBi. This could be attributed to the larger band in this matrial. The electronic structure of LaPdBi hosts 8 triple points with *single* band-inversion at 7% tensile strain (shown in Figure 1(g-h)). However, the tensile strain required for achieving 24 triple points was observed to be unrealistically large, and therefore not reported here. In conclusion, we observe, both ScPdBi and LaPdBi obey the phase diagram (Figure 4(d)) while traversing through different band inversions.

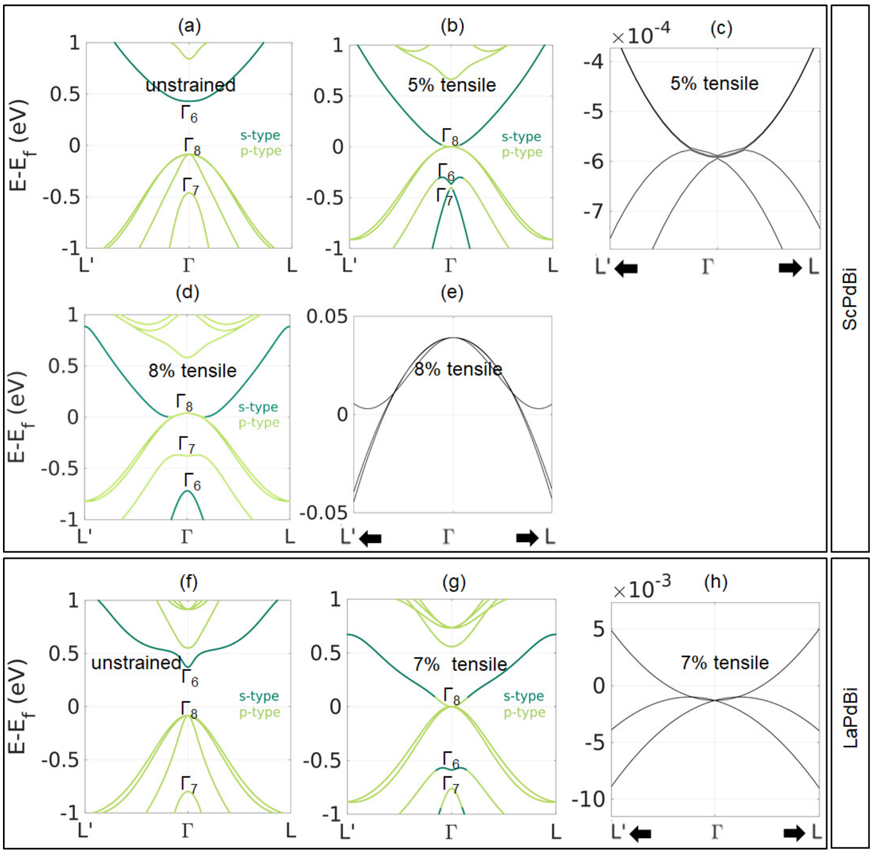

Figure 1: (a-e) Band inversions and triple points in ScPdBi under tensile strain. (c,e) The zoomed view of triple points around Γ point in ScPdBi. (f-h) Band inversions and triple points in LaPdBi under tensile strain.

Band inversion and triple points in YPtBi, LuPtBi and LuPdBi through compressive strain

The LuPdBi, YPtBi and LuPtBi host six triple points along each [111] direction with *two* band inversions at unstrained state (Figure 2(a-b,f,j)). As we observe from the Figure 2, at 2% compressive strain, LuPdBi just reaches the *single* band inversion zone with 8 triple points in the Brillouin zone. Application of further compressive strains opens up a band gap at Γ point. This semiconducting state in LuPdBi at 5% compressive strain is shown in the Figure 2(e). The similar trend of transitions is also observed for YPtBi and LuPtBi materials. At 3% and 5% compressive strains, respectively, these attain *single* band inversion with 8 triple points in the Brillouin zone. Then, as shown in Figure 2(i,m), both of these materials exhibit a gapped electronic structure at 7% and 9% compressive strains, respectively.

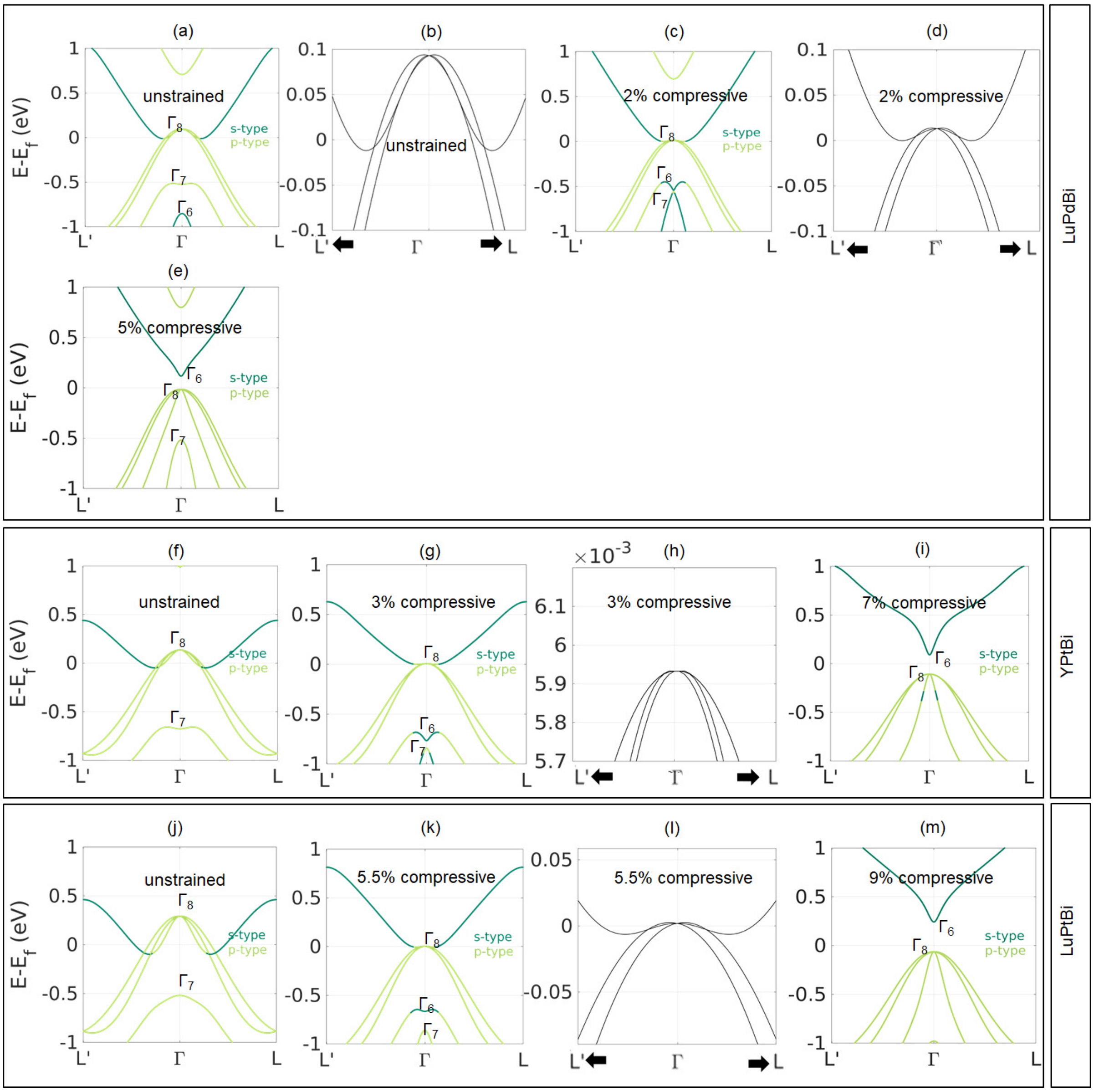

Figure 2: Band inversions and triple points in LuPdbi (a-e), YPtBi (f-i) and LuPtBi (j-m) under compressive strains.

As LuPdBi is more susceptible to achieve band-inversions under compressive strain, we focus on this material to examine the mechanism associated with band inversions. In Figure 3(a), we have shown the variation of Γ_8, Γ_7, Γ_6 and Fermi energy as a function of compressive strain. Here, we observe an opposite trend than YPdBi under tensile strain. The energy associated with Γ_6 rises much faster to facilitate LuPdBi achieve different band-inversions. Similarly, the change in the occupation of various orbitals in LuPdBi (Figure 3(b)) shows that the occupation of *s*-orbital in Bi decreases with compressive strain. This is in

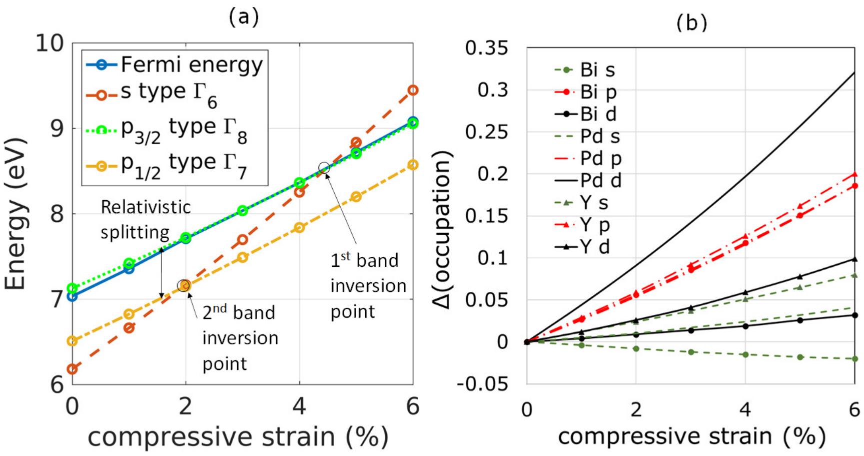

Figure 3: (a) The change in Fermi energy and the energies of Γ_6, Γ_8 and Γ_7 with compressive strain in LuPdBi. (b) The change in the occupation of various orbitals with compressive strain.

contrast to the trend observed in YPdBi.

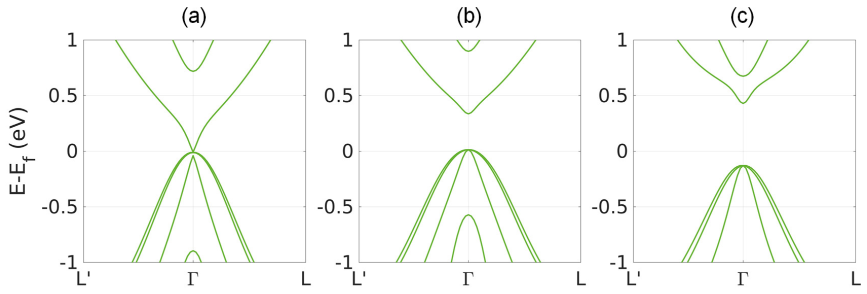

Figure 4: HSE bandstructure using GGA optimized lattice parameters for YPdBi (a), ScPdBi (b) and LaPdBi (c).

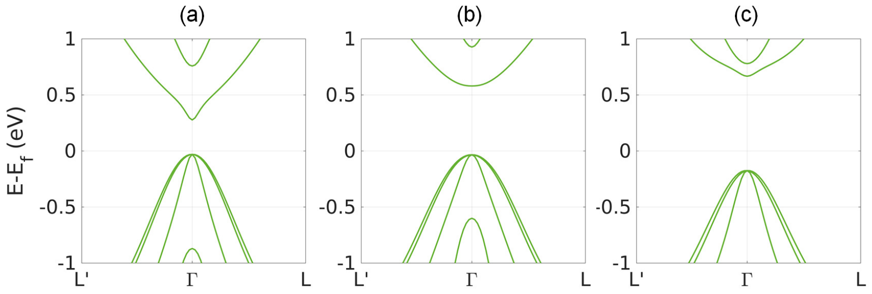

Figure 5: HSE bandstructure using experimental lattice parameters for YPdBi (a), ScPdBi (b) and LaPdBi (c).

HSE06 band-structures

In order to validate LDA calculated band-gaps and accompanying strains to induce band-inversions for materials studied in the present work, we calculated the HSE06 band-structures for YPdBi, ScPdBi and LaPdBi. Both GGA optimized and experimental lattice parameters were used in the calculations. From our calculations we find that the use of experimental lattice parameters with HSE06 functional overestimates the band gaps (0.31 eV, 0.61 eV, 0.84 eV, respectively for YPdBi, ScPdBi and LaPdBi as shown in Figure 5) whereas, the HSE06 functional with GGA optimized lattice parameters wrongly predicts the band-inverted metallic nature for YPdBi by underestimating the band gap (see the Figure 4). We further observe that

with HSE06, approximately 3% tensile strain over experimental lattice parameters is required to obtain triple point semimetallic state. Since there is no experimental data on the band-gaps for these materials, the only way to validate our results is to compare them against reported metallic or semiconducting nature. It should also be noted that HSE06 functional with experimental lattice parameter predicts similar band-gaps to MBJLDA,[1] which is proven to predict larger band-gaps than experiments in half Heusler and half-metallic Heusler alloys.[2,3]

References

[1] Wael Al-Sawai, Hsin Lin, RS Markiewicz, LA Wray, Y Xia, S-Y Xu, MZ Hasan, and A Bansil. Topological electronic structure in half-heusler topological insulators. *Physical Review B*, 82(12):125208, 2010.

[2] Markus Meinert. Modified becke-johnson potential investigation of half-metallic heusler compounds. *Physical Review B*, 87(4):045103, 2013.

[3] Hangtian Zhu, Jun Mao, Yuwei Li, Jifeng Sun, Yumei Wang, Qing Zhu, Guannan Li, Qichen Song, Jiawei Zhou, Yuhao Fu, et al. Discovery of TaFeSb-based half-Heuslers with high thermoelectric performance. *Nature communications*, 10(1):1–8, 2019.
